# Prediction of small for size syndrome after extended hepatectomy: Tissue characterization by relaxometry, diffusion weighted magnetic resonance imaging and magnetization transfer

**DOI:** 10.1371/journal.pone.0192847

**Published:** 2018-02-14

**Authors:** Christian Eberhardt, Moritz C. Wurnig, Andrea Wirsching, Cristina Rossi, Idana Feldmane, Mickael Lesurtel, Andreas Boss

**Affiliations:** 1 Institute of Diagnostic and Interventional Radiology, University Hospital Zurich, Zurich, Switzerland; 2 Swiss Hepato-Pancreatico-Biliary and Transplantation Center, Department of Surgery, University Hospital Zurich, Zurich, Switzerland; 3 Department of Digestive Surgery and Liver Transplantation, Croix-Rousse University Hospital, Lyon, France; University Hospital Oldenburg, GERMANY

## Abstract

This study aimed to monitor the course of liver regeneration by multiparametric magnetic-resonance imaging (MRI) after partial liver resection characterizing Small-for-Size Syndrome (SFSS), which frequently leads to fatal post-hepatectomy liver failure (PLF).

Twenty-two C57BL/6 mice underwent either conventional 70% partial hepatectomy (cPH), extended 86% partial hepatectomy (ePH) or SHAM operation. Subsequent MRI scans on days 0, 1, 2, 3, 5 and 7 in a 4.7T MR Scanner quantified longitudinal and transverse relaxation times, apparent diffusion coefficient (ADC) and the magnetization-transfer ratio (MTR) of the regenerating liver parenchyma. Histological examination was performed by hematoxylin-eosin staining. After hepatectomy, an increase of T1 time was detected being larger for ePH on day 1: 18% for cPH vs. 40% for ePH and on day 2: 24% for cPH vs. 49% for ePH. An increase in T2 time, again greater in ePH was most pronounced on day 5: 21% for cPH vs. 41% for ePH. ADC and MTR showed a decrease on day 1: 21% for ePH vs. 13% for cPH for ADC, 15% for ePH vs. 11% for cPH for MTR. Subsequently, all MR parameters converged towards initial values in surviving animals. Dying PLF animals exhibited the strongest increase of T1 relaxation time and most prominent decreases of ADC and MTR. The retrieved MRI biomarkers indicate SFSS and potentially developing PLF at an early stage, likely reflecting cellular hypertrophy with extended water content and concomitantly diluted cellular components as features of liver regeneration, appearing more intense in ePH as compared to cPH.

## Introduction

Liver resection is often the only chance of cure in patients with liver malignancies such as hepatocellular carcinoma or colorectal liver metastases [1]. In the last years, techniques of liver resection, also termed as partial hepatectomy (PH), have been significantly refined with an increasing number of patients being amenable to curative resection [2]. In case of extended PH (>70% resected liver volume) patients may develop postoperative, often fatal complications such as post-hepatectomy liver failure (PLF) due to insufficient volume or insufficient function of the remnant liver, also known as small for size syndrome (SFSS). The incidence of PLF after extensive PH is estimated to reach up to 9% with a mortality of up to 5% [3]. SFSS is not uniformly defined and the underlying molecular mechanisms are not fully understood [1]. The prospect of liver recovery after PH is therefore difficult to judge and the currently applied prognostic criteria diagnosing SFSS and predicting its exacerbation towards PLF are mainly based on different grades of hepatic encephalopathy and on blood parameters such as prolonged prothrombin time, hyperbilirubinemia and hypoalbuminemia determined on consecutive postoperative days (PODs) [1, 3].

Magnetic-resonance imaging (MRI) is typically applied for pre-operative planning of the treatment strategy, mostly to diagnose the type of tumors, compute liver volume and predict the size of the liver remnants [4]. However, magnetic resonance provides not only excellent intrinsic soft-tissue contrast for 3D liver segmentation but offers also a variety of imaging biomarkers allowing for a deeper understanding of the underlying pathophysiology. The tissue magnetic relaxation times, T1 and T2, reflect various factors such as macromolecular tissue composition, as well blood and water content, and are therefore fundamental for the contrast seen in an MRI examination. In the last years, diffusion-weighted imaging has become increasingly important in liver imaging, as the restriction of passive water diffusion described by apparent diffusion coefficient (ADC) maps may be indicative of a malignant liver tumor [5]. The magnetization transfer is a further contrast, which is independent of the classical relaxation properties, measuring the interaction of water-molecules with the macromolecular environment, typically described by the magnetization-transfer ratio (MTR).

The elucidation of the inherent mechanisms of SFSS and PLF as well as the development of non-invasive molecular imaging techniques to monitor the early post-operative patient recovery and to diagnose impending SFSS might be of clinical importance. The purpose of the present study was twofold: (a) to characterize SFSS and (b) to provide early discriminators for SFSS and its developing complications such as PLF using non-invasive multimodal MRI techniques in mouse models of conventional partial hepatectomy (cPH) and extended partial hepatectomy (ePH), the latter representing the animal model of SFSS.

## Material and methods

### Animal protocol and study design

The study on C57Bl/6 mice was approved by the Veterinary Office of the Canton Zurich (license no. 131/2011). Mice (n = 57; 25–32 g) at the age of 8–10 weeks were housed in the animal facility of our hospital with a 12-hour day-night cycle. All animals received care in strict accordance to The Principles of Laboratory Animal Care (promulgated in 1985 and most recently revised in 1996). Animals were kept under standardized conditions in accordance with the institutional animal care guidelines with unlimited access to standard diet and water. Research staff conducting the experiments has received special education and training accredited by the Federation of Laboratory Animal Science Associations (FELASA).

**Fig 1:**
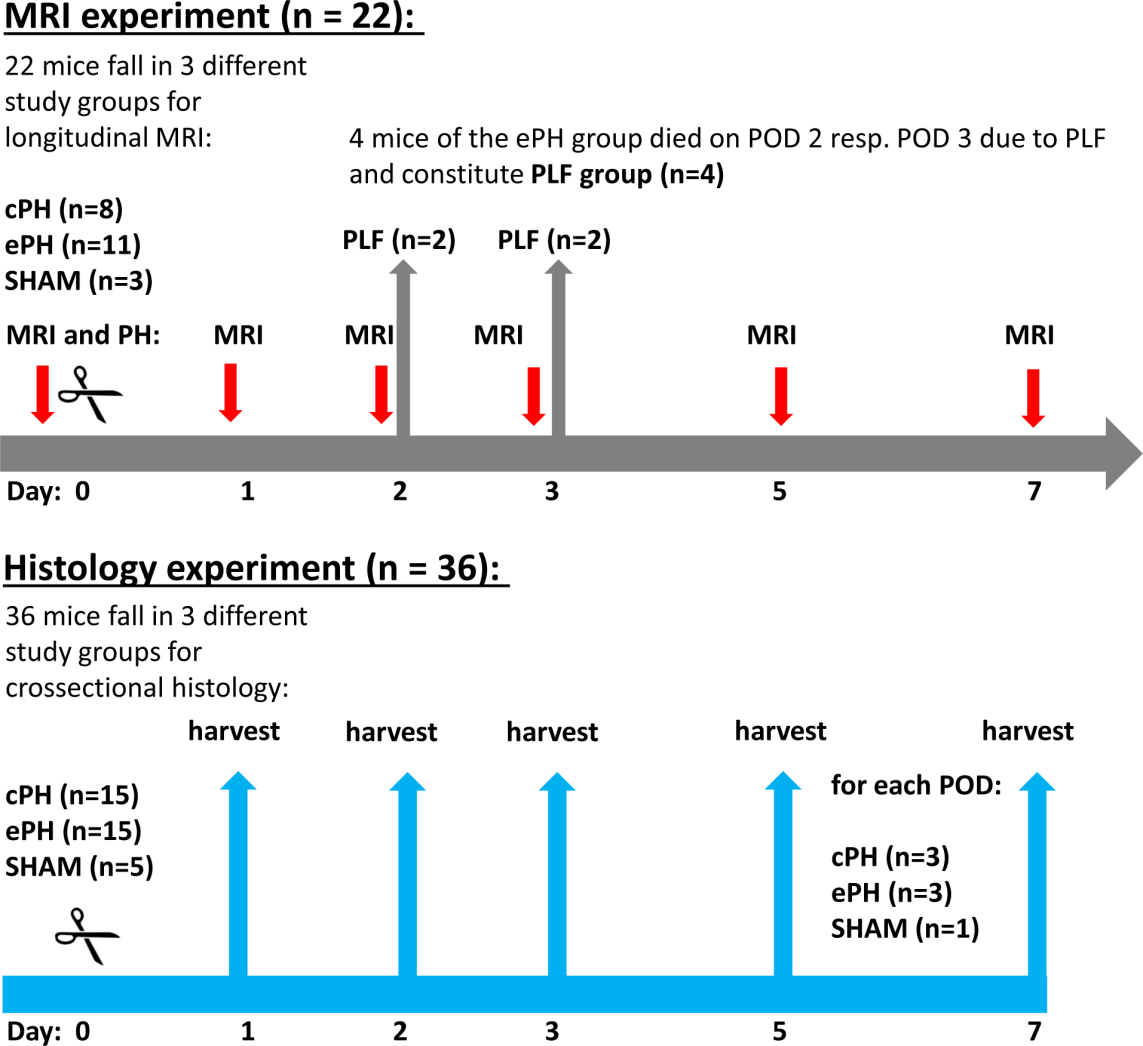
Study flowchart of all animals assigned to assigned either MRI experiments or histology. The study design comprised essentially two cohorts of mice. One cohort of mice was dedicated to MRI assessment after cPH, ePH or SHAM operation in a longitudinal study (nMRI = 22), the other cohort, requiring tissue harvest for histological assessment of the liver parenchyma corresponding to each time point of the MRI investigation was examined in a cross-sectional study after cPH, ePH or SHAM operation (nHistology = 36).

The study design comprised essentially two cohorts of mice. One cohort of mice was dedicated to MRI assessment after cPH, ePH or SHAM operation in a longitudinal study (n_MRI_ = 22), the other cohort, requiring tissue harvest for histological examination of the liver parenchyma was hence examined in a cross-sectional study after cPH, ePH or SHAM operation (n_Histology_ = 36), providing essentially the histology of liver regeneration corresponding to each time point of the MRI investigation. **Fig 1**depicts the study flowchart of the entire cohort.

### Surgery

#### Surgical anaesthesia, analgesia and postoperative care

For analgesia, 0.1 mg/kg bodyweight (BW) buprenorphine (Temgesic, Indivior, UK) was administered subcutaneously 30 min prior start of any surgical intervention. Subsequently, anaesthesia was induced by 5% (v/v) isoflurane supplemented oxygen (Attane, Minrad I, Buffalo, NY) in an enclosed container. After applying eye ointment and shaving of the abdomen, animals were fixed for surgical intervention.

During surgical intervention, anaesthesia was maintained with 2–4% (v/v) isoflurane supplemented oxygen administration via small nose cone. Anaesthetic depth was monitored by clinical parameters such as respiratory rate and inhalation depth, colour of mucous membranes and inner organs and was adjusted accordingly by the isoflurane concentration. Having completed the surgical procedure, animals recovered in a warmed cage and if needed, buprenorphine (0.1 mg/kg BW, s.c.) was readministered within 3 hrs, thereafter analgesia with buprenorphine (0.1 mg/kg BW, s.c.) was repeated every 8 hrs on POD 1 and 2.

Afterwards, the animals were carefuly monitored at least once a day for the duration of the experiment i.e. until POD 7 for the cohort undergoing MRI (n_MRI_ = 22) examination respectively until POD 1, 2, 3, 5, 7 (n_Histology_ = 35) for the cohort dedicated to tissue harvest for histological examination. However, signs of moderate to severe clinical pain, distress and discomfort unalleviated by analgesia, weight loss of ≥ 15% of original body weight, mutilation of the site of operation, jaundice and behavioral changes resulted immediately to a premature termination of the experiment.

#### Conventional 70% Partial Hepatectomy (cPH)

After a midline laparotomy on mice undergoing 70% cPH (n_MRI_ = 8, n_Histology_ = 15), medial and left lateral liver lobes were dissected out of their ligaments. Then, the cystic duct was ligated with 8–0 polypropylen suture (Prolene, Ethicon, Cincinnati, OH, USA) and the gallbladder removed. Subsequently, the left and right medial lobes and the left lateral lobe were ligated with 6–0 silk suture (Look SP114 6/0 Silk Spool, Hospeq, Miami, FL, USA) and resected. The remnant liver part corresponded to the right and caudate lobes. Finally, the abdomen was closed with interrupted 5–0 polypropylen sutures (Prolene, Ethicon).

#### Extended 86% Partial Hepatectomy (ePH) and Post-Hepatectomy Liver Failure (PLF)

After a midline laparotomy on mice undergoing 86% ePH (n_MRI_ = 11) (n_Histology_ = 15), the liver was freed from ligaments and portal tributaries to the medial, right inferior, left and caudate lobes were ligated individually with 8–0 polypropylen sutures (Prolene, Ethicon). Cholecystectomy was performed after ligation of the cystic duct with 8–0 polypropylen sutures (Prolene, Ethicon). Subsequently, the deportalized liver lobes were ligated with 6–0 silk suture (Look SP114 6/0 Silk Spool, Hospeq) and resected. The remnant liver part corresponded to the right upper lobe. The abdomen was closed with interrupted 5–0 polypropylen sutures (Prolene, Ethicon). However, 4 animals died due to PLF (n_MRI_ = 4) (n_MRI_ = 2 death on day 2, n_MRI_ = 2 death on day 3) which constituted the PLF group.

Taken together, animals after 86% ePH constituted two study groups, namely the ePH group (n_MRI_ = 7), representing SFSS and the PLF group (n_MRI_ = 4), suffering a fatal PLF due to SFSS **(S1 Fig)**.

#### SHAM operation (n = 3)

Mice (n_MRI_ = 3) (n_Histology_ = 5) underwent a midline laparotomy. Subsequently, medial- and left lateral liver lobes were freed from ligaments and the abdomen was closed with interrupted 5–0 polypropylen sutures (Prolene, Ethicon).

### Histology

A separate cohort of mice (n_Histology_ = 35) was sacrificed for histological examination of the liver remnant on PODs 1, 2, 3, 5 and 7 after cPH respectively ePH with three animals for each time point, and after SHAM with one animal for each time point. Liver tissue samples of the right lobe were immersion fixed in 4% buffered formalin (24 h, 20°C), dehydrated through a series of graded alcohols, cleared in Histo Clear (Brunschwig, Basel, Switzerland), impregnated with liquid wax (Paraplast, Leica Biosystems, Muttenz, Switzerland) and cut into 3 μm tissue sections. Specimens were subsequently stained with hematoxylin-eosin (H&E). For immunohistochemistry against phosphorylated Histone 3 (pH3), sections were transferred to Target Retrieval Solution (K8004, Dako Denmark A/S, Glostrup, Denmark, 20 min, 97°C) within a Dako PT Link (PT100/PT101, Dako Denmark A/S) for the 3-in-1 procedure i.e. deparaffinization, rehydration, and heat-induced epitope-retrieval (HIER) of formalin-fixed, paraffin-embedded tissue sections. A standard IHC staining protocol was performed on a Dako Autostainer Link48 Instrument (Dako Denmark A/S) for pH3 using a rabbit polyclonal anti-pH3 antibody (Anti-H3S10p, 06–570, Merck Millipore, Germany) working dilution 1:50 in Dako Antibody Diluent (S2022, Dako Denmark A/S, 20min, 20°C). (06–570, Merck Millipore, Germany). The visualization system consisted of the Dako EnVision™ Rabbit/HRP/DAB system and Hematoxylin as counterstain. After IHC staining the tissue specimens were dehydrated, permanently mounted and microscopically evaluated.

### Magnetic resonance imaging

The animals underwent MRI scans measuring longitudinal and transverse relaxation times, diffusion properties and magnetization transfer ratio. The liver regeneration was further assessed by MR liver volumetry. For that purpose, mice were placed in prone position on a respiratory sensor (SA Instruments, Stony Brook, NY, USA) located in a plastic holder with nose cone, providing air supplemented with 1.0–1.5% isoflurane (Attane, Minrad I, Buffalo, NY), and covered by a warming pad to maintain the body temperature. Ophtalmic ointment was used averting harmful dryness of the eyes while scanning. Experiments were performed on a 4.7T small animal MRI system (Pharmascan 47/16 US; Bruker BioSpin MRI GmbH, Ettlingen, Germany) with a gradient strength of 375 mT/m and a slew rate of 3375 T/m/s equipped with a linear polarized hydrogen whole-body mouse transmit-receive radiofrequency coil. The measurements were performed on the day before surgery and then on PODs 1, 2, 3, 5 and 7.

After a gradient-echo localizer scan in three directions, the imaging protocol included an axial and sagittal T2-weighted, two-dimensional fast spin-echo sequences (2D-RARE) with time-to-echo (TE) = 33ms/ time-to-repetition (TR) = 2500 ms, number of averages (AVG) = 1, receiver bandwidth (BW) = 50000 Hz, echo train length = 8, echo spacing = 11.0 ms, field of view (FoV) = 40 x 40 mm, slice thickness = 1 mm, matrix 256 x 256, voxel size = 0.156 mm x 0.156 mm x 1 mm were recorded to cover the entire abdomen in 20 slices. The duration of the scan was 1 min 20 sec. All settings of the following MRI experiments are also listed in **S1 Table**.

#### Liver volumetry

For volumetric assessment of liver regeneration, a three-dimensional fast low angle shot magnetic resonance imaging (3D-FLASH) sequence was applied with following settings: TE = 2.6 ms, TR = 15.0 ms, AVG = 4, flip angle (FA) = 20°, BW = 100 000 Hz, FoV 30 x 30 mm, slice thickness = 22.5 mm, acquisition matrix 256 x 256 x 96, voxel size 0.117 mm x 0.117 mm x 0.234 mm, scan time 20min 21s.

#### Relaxometry

Longitudinal relaxation times (T1 times) were measured using a respiratory triggered two-dimensional segmented inversion-recovery true fast imaging with steady precession (2D-trueFISP) sequence with TE = 2.25 ms, TR = 4.5 ms, n = 16 segments resulting in 60 inversion times TI starting with TI_1_ = 90 ms and TI_n_ = TI_1_ + n*144ms, FA = 60°, AVG = 4, BW = 81521.7 Hz, echo spacing = 2.2 ms, FoV = 30 x 30 mm, slices = 1, slice thickness = 1.5 mm, matrix = 128 x 128, voxel size = 0.234 mm x 0.234 x 1.5 mm. Scan duration was calculated with approximately 10 min 40s, but slightly deviated due to respiratory triggering.

Transverse relaxation times (T2 times) were determined by a 2D-RARE sequence with 5 different echo times and 7 repetition times: TE = 11, 33, 55, 77 and 99 ms, TR 118, 258, 400, 800, 1500, 3000 and 4000 ms, RARE factor = 2, AVG = 2, BW = 480769 Hz, FoV 30 x 30 mm, slices = 10, slice thickness = 1.5 mm, matrix size 128 x 128, voxel size = 0.234 cm x 0.234 cm x 1.5mm. The acquisition time was 7 min 12 sec. Due to technical problems, one 86% PH animal could not be measured on day 5 using this sequence, hence opted out of the study and therefore T2 relaxation was assessed on 86% PH with n_MRI_ = 6.

#### Diffusivity

Water diffusion was assessed using a respiratory triggered diffusion-weighted spin-echo echo-planar imaging sequence with fat saturation and the following settings: TE = 30 ms, TR = 3000 ms, AVG = 8, fat suppression prepulse, BW = 250 000 Hz, number of b-values 2 (0, 800 s/mm^2^), field of view (FoV) 30 x 30 mm, slices = 10, slice thickness = 1.5 mm, matrix size 128 x 128, voxel size = 0.234 x 02.34 x 1.5 mm. Scan duration was calculated with approximately to 10 min 40s, but deviated due to respiratory triggering.

#### Magnetization transfer

Magnetization-transfer (MT) was measured with two three-dimensional spoiled gradient-echo sequences (3D-FLASH). Sequence parameters were: TE = 2.65 ms, TR = 18.4 ms, AVG = 8, FA = 12°; BW = 100 000 Hz, FoV 30 x 30 mm, slice thickness = 15 mm, acquisition matrix 128 x 128 x 10, voxel size = 0.234 mm x 0.234 mm x 1.5 mm. The scan time amounted to 2 min 21 sec. This sequence was acquired with a MT pre-pulse (Gaussian pulse shape applied for each TR, off-resonance frequency 1500 Hz, flip angle 1200°, magnetization transfer pulse length = 10.96 ms, magnetization transfer pulse BW = 250 Hz, interpulse delay 0.01 ms, no dummy pulses). A reference scan for MTR quantification was acquired using the same sequence without the MT prepulse.

### Quantification of MR imaging biomarkers

For all fitting procedures, non-linear least square fits to the signal intensities were performed based on the Levenberg-Marquardt-Algorithm (Matlab “lsqcurvefit”) in a pixel-by-pixel manner.

For calculation of T1 time, the signal intensities of the inversion-recovery trueFISP sequence were fitted to the equation

$$S({TI}_{i})=S_{0}(1-INV*e^{-\frac{{TI}_{i}}{T1*}})$$ (1)

with INV indicating the ratio between the initial signal S_0_ and the steady state signal and T_1_ being calculated from

$$T_{1}=T_{1}^{*}cos\frac{\alpha }{2}(INV-1)$$ (2)

according to a Schmitt et al. [6]

The T2 time was calculated from the RARE sequence by fitting the equation

$$S({TE}_{i})=S(0)*e^{-\frac{{TE}_{i}}{T2}}+N$$ (3)

to the signal intensities of the different echo time TE_i_ with N meaning the noise.

The apparent diffusion coefficient (ADC) was calculated from the logarithmic signal intensities

$$ADC=\frac{1}{b}\;ln\;\left(\frac{S(b_{0})}{S(b)}\right)$$ (4)

with S(b) meaning the signal intensity at the b-value of 800 s/mm^2^, and b_0_ the b-value = 0 s/mm^2^.

The MTR values (in %) were calculated from the MT sequences with and without MT prepulse in the following manner:

$$MTR=\frac{M_{0}-M_{sat}}{M_{0}}$$ (5)

with M_0_ meaning the signal intensity without MT prepulse and M_sat_ the signal intensity with MT prepulse.

### Definition of a region-of-interest (ROI)

**Fig 2:**
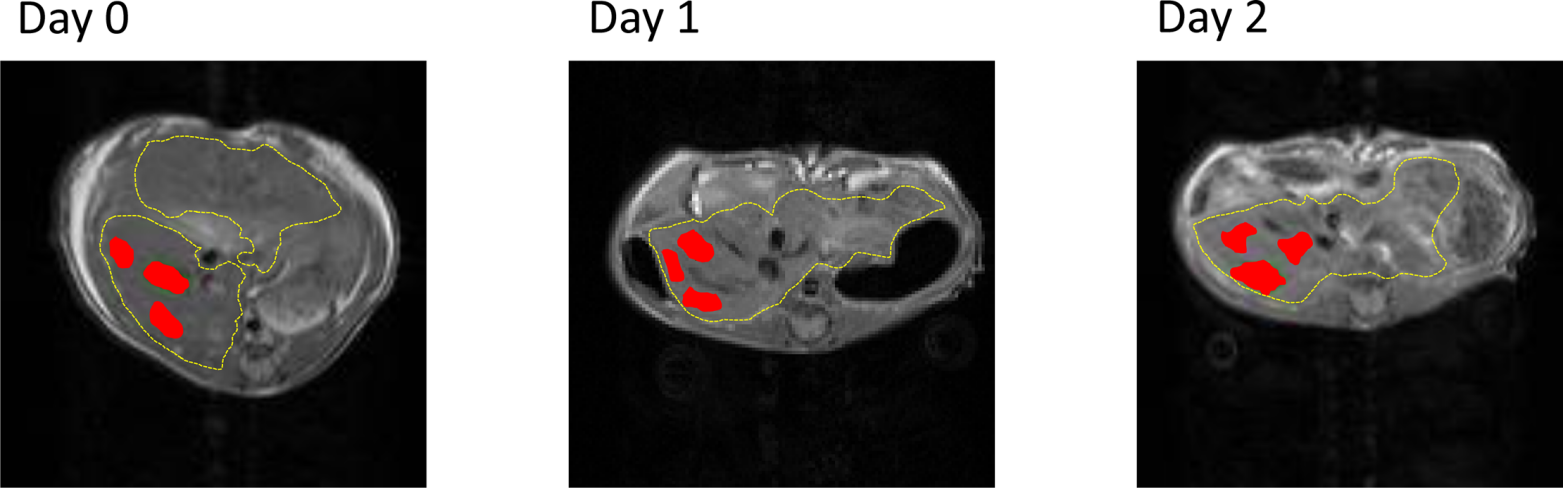
Typical ROI drawing is depicted as red areas. Three independent polygonal ROIs were drawn in the right lobe under avoidance of large macroscopic vessels. The contour of the liver is indicated by a yellow line.

Applying custom-written Matlab scripts (MathWorks, Natick, MA, USA) a region-of-interest (ROI) analysis was performed with manually drawn RoIs on anatomical images of the respective sequences and subsequently copied to the parametrical maps. Three independent polygonal ROIs were drawn in the right lobe under avoidance of large vessel structures within the parenchyma and representative ROIs are shown in **Fig 2**. The image noise was determined in a ROI positioned outside the body in the upper left hand corner in the background of the image and corrected by squared subtraction according to Gudbjartsson [7].

For the volumetric assessment of liver regeneration, the liver contour was manually retraced on each of the 0.23mm-thick slices using ImageJ (http://imagej.nih.gov/) [8]. The sum of all liver areas multiplied by the slice thickness retrieved the liver volume. The liver volume was normalized to its original volume prior surgery and is therefore presented as relative liver volume in percent.

### Statistical evaluation and image post-processing

For descriptive analysis, mean values and standard deviations of the liver remnant volume, the T1 and T2 times, ADC values and MTR were calculated and numeric data are presented as mean ± standard deviation. Statistical evaluation of the acquired parameters between the study groups was performed by two-way analysis of variance (ANOVA) with Bonferroni correction using GraphPad Prism 5 software (GraphPad Software, Inc., La Jolla, CA, USA). Assessing the correlation between the liver volume and the retrieved MR parameters for the regenerating liver, a Pearson correlation was carried out using SPSS Software vers. 25 (IBM Corporation, Armonk, NY, USA) and GraphPad Prism 5. Generally, a P-value of less than 0.05 was considered significant. All MR parameters obtained for animals after ePH have been further analyzed by binomial logistic regression accommodating the MR parameters as quantitative, independent and predictive variables and the death of the animal by PLF as dependent dichotomous variable by using SPSS.

## Results

### Extended hepatectomy is associated to cellular hypertrophy and lipid accumulation

**Fig 3:**
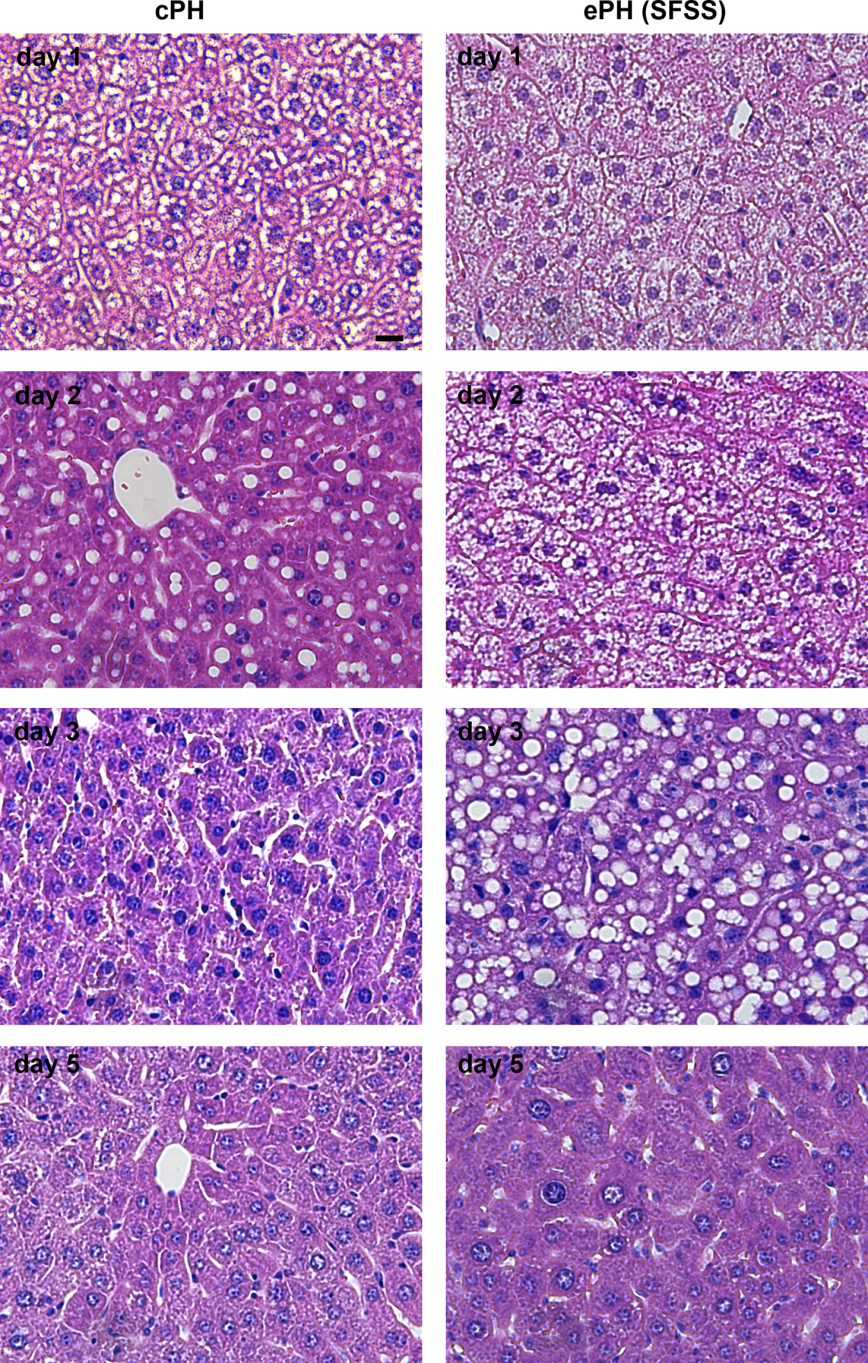
Representative images depicting H&E staining of regenerating liver parenchyma. Hematoxylin stained nuclei appear blue, whereas eosin stains proteins nonspecifically pink. The image shows formalin-fixed, paraffin embedded tissue sections of liver parenchyma after cPH on POD 1, 2, 3 and 5 (left panel) and parenchyma after ePH on POD 1, 2, 3 and 5 (right panel). The reported cellular hypertrophy leading to the early volume gain of the regenerating liver is mainly driven by an increased accumulation of fluids and lipids into parenchymal cells, resulting in the vacuolated appearance during the first few days [9]. For all images, scale bar is 20 μm.

On representative H&E stains of paraffin embedded tissue sections, there are notable morphological alterations over the time course of liver regeneration, but also within the two study groups, cPH and ePH on PODs 1, 2, 3, and 5 **(Fig 3)**. Throughout the process of early liver regeneration, the liver parenchyma appears homogenous without any visible signs of necrosis. Following hepatectomy, hepatocytic cytoplasm in the liver remnant appears swollen, indicating a cellular hypertrophy in both groups, due to a notable infiltration of fluids and lipids in the hepatocytes. This phenomenon is most pronounced on day 1 after cPH and on days 1 and 2 after ePH **(Fig 3)**. Subsequently, the steatotic, vacuolated structure of lipid droplets within hepatocytes remains visible on day 2 after cPH and on day 3 after ePH **(Fig 3)**. The representative immunohistochemical images depict already pH3 positive hepatocytes on POD 2 for cPH, but no positive hepatocytes for ePH at this time point and in contrast to cPH, the number of positive, thus mitotic hepatocytes is much higher in the ePH group on POD3 **(S2 Fig)**. There are hardly any positive hepatocytes detectable in the SHAM operated liver parenchyma **(S3 Fig)**.

### Liver remnant volume is markedly decreased after ePH and PFL compared to cPH

**Fig 4:**
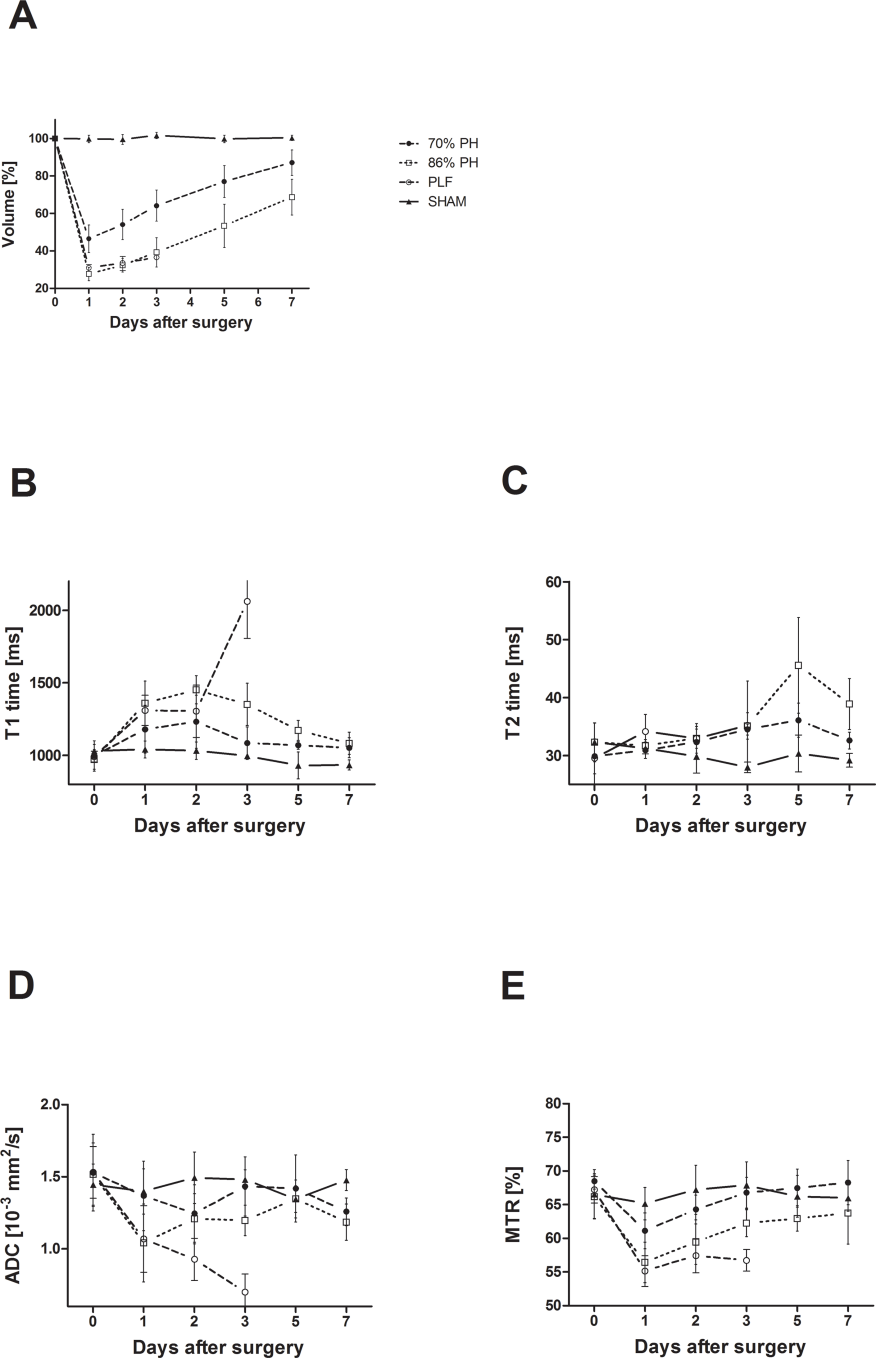
Liver regeneration assessed by multimodal MRI for animals having undergone partial hepatectomy or SHAM surgery. The diagrams depict the measurements of MR volumetry (A), longitudinal (B) and transverse relaxation (C), diffusion (D) and magnetization transfer (E) of the regenerating liver remnant. After its initial liver volume loss, the liver could already replenish liver parenchyma up to 50% for cPH and up to 30% for ePH one day after hepatectomy (A). T1 times, lesser the T2 times, increase after hepatectomy dependent on the extent of resection with almost complete recovery to baseline if the animal survives (B, C). There are further remarkable decreases in diffusion and magnetization transfer dependent on extent of resection (D, E). These observations appear plausible considering the reported hypertrophy of parenchymal cells, caused by an elevated lipid and fluid content early after resection. The cellular increase of water presumably prolongs the T1 relaxation, the cellular hypertrophy restricts diffusion whereas the cellular increase of water and lipids hampers magnetization transfer.

When compared to the SHAM group, MR liver volumetry showed a decreased liver remnant volume after hepatectomy in all other groups (cPH, ePH and PLF). Compared amongst each other, all liver resection study groups, exempt ePH vs. PLF, showed significant differences concerning the postoperative liver volumes (cPH vs. ePH and ePH vs. SHAM day 1—day 7 P<0.001; cPH vs. SHAM day 1—day 5 P<0.001, day 7 P < 0.05). Already on POD 1, liver remnant volume as percentage of preoperative total liver volume reached 46,4% ± 7,3% after cPH, 27,7% ± 3,6% after ePH % and 30,9% ± 1,3% for the PFL group **(Table 1)**. On POD 2, the liver further gained 54,0% ± 8,1% after cPH, 32,3% ± 3,6% after ePH, and 33,5% ± 3,1% for the PFL group. Finally, liver remnant volumes on POD 7, represented 87,1% ± 6,8% after cPH and 68,7% ± 9,6% after ePH **(Fig 4A; Table 1).**

**Table 1:** Liver regeneration was monitored by MR volumetry and by longitudinal T1 and transversal T2 relaxometry for all study groups, cPH, ePH, PLF and SHAM, prior surgery and on POD 1, 2, 3, 5, 7.

Day	Volumetry [%]				Longitudinal relaxation T1 [ms]				Transversal relaxation T2 [ms]			
	cPH	ePH	PLF	SHAM	cPH	ePH	PLF	SHAM	cPH	ePH	PLF	SHAM
0	100.0±0.0	100.0± 0.0	100.0±0.0	100.0±0.0	997.0±104.8	972.6± 67.7	991.7± 38.0	1030.1±46.0	29.9±3.0	32.3±3.4	29.5±6.1	32.3±0.5
1	46.4±7.3	27.7± 3.6	30.9±1.3	99.8± 2.0	1180.0±165.1	1359.7±152.6	1310.4±103.4	1040.4±58.4	30.9±1.4	31.7±1.0	34.1±3.0	31.2±1.0
2	54.0±8.1	32.3± 3.6	33.5±3.1	99.4±2.6	1232.2±182.1	1451.7± 97.4	1304.2±180.0	1033.1±60.8	32.3±2.7	33.0±2.6	32.9±1.6	29.8±2.9
3	64.1±8.3	39.3± 7.8	36.4±0.9	101.6±1.6	1084.8±112.6	1351.7±144.8	2060.6±254.8	996.9± 5.4	34.5±1.0	35.1±2.3	35.2±7.6	28.0±0.9
5	77.0±8.5	53.3±11.6	n/a	99.8±2.0	1069.8± 26.1	1172.6± 68.8	n/a	930.5±92.5	36.1±2.9	45.6±8.3	n/a	30.4±3.2
7	87.1±6.8	68.7± 9.6	n/a	100.3±1.5	1053.1± 69.2	1083.4± 76.7	n/a	934.1±34.6	32.6±1.5	38.9±4.4	n/a	29.2±1.2

### Quantitative multimodal MRI differentiates between liver tissue after cPH and ePH

On day 1 after resection, an increase of T1 relaxation time was detected being generally more pronounced after ePH. T1 relaxation increased on POD 1 by 18% for cPH vs. 40% for ePH and on POD 2 respectively by 24% for cPH vs. 49% for ePH animals **(Fig 4B; Table 1)**. There was a prolongation in T2 relaxation, again greater after ePH, with a prominent increase on day 5 by 21% for cPH vs. 41% for ePH **(Fig 4C; Table 1)**. ADC and MTR showed a strong decrease after ePH on day 1 with 21% for ePH vs. 13% for cPH for ADC and with 15% for ePH vs. 11% for cPH for MTR **(Fig 4D and 4E; Table 2)**. After having reached their maximum (T1 and T2), respectively their minimum (ADC and MTR), all MR parameters converged towards their initial value on POD 7 in surviving animals. In the PLF animals, which died between day 2 and 3 after extended hepatectomy, a strong increase of T1 time and a very prominent decline of ADC and MTR were observed **(Fig 4A**–**4D; Tables 1 and 2)**.

**Table 2:** Liver regeneration was monitored by diffusion-weighted MRI and magnetization transfer MRI for all study groups, cPH, ePH, PLF and SHAM, prior surgery and on POD 1, 2, 3, 5, 7.

Day	ADC [10^−3^ mm^2^/s]				MTR [%]			
	cPH	ePH	PLF	SHAM	cPH	ePH	PLF	SHAM
0	1.54±0.25	1.52±0.22	1.53±0.18	1.45±0.14	68.5±1.2	66.2±3.2	67.2±2.0	66.5±3.7
1	1.34±0.24	1.04±0.27	1.07±0.23	1.40±0.16	61.1±2.7	56.4±3.0	55.1±2.3	65.1±2.4
2	1.24±0.18	1.21±0.17	0.93±0.15	1.49±0.18	64.3±2.1	59.4±3.3	57.4±2.6	67.2±3.6
3	1.42±0.19	1.20±0.11	0.70±0.13	1.48±0.07	66.8±2.3	62.2±2.0	56.7±1.6	67.9±3.5
5	1.42±0.22	1.35±0.13	n/a	1.34±0.09	67.5±2.8	62.9±1.9	n/a	66.2±3.1
7	1.27±0.09	1.19±0.13	n/a	1.48±0.07	68.3±3.3	63.7±4.6	n/a	66.0±2.0

**Fig 5:**
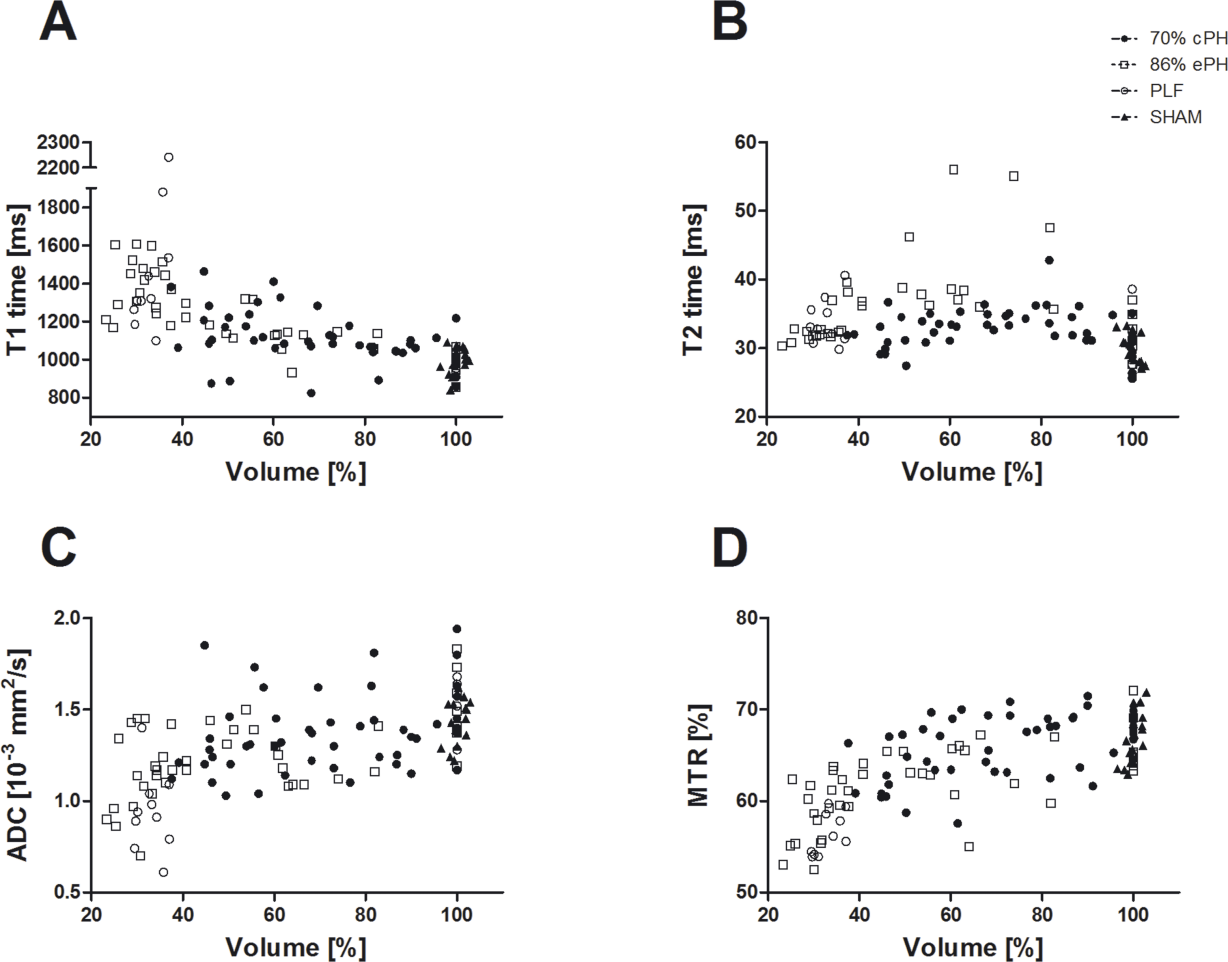
Correlation of the measured MRI parameters with the volume regeneration of the hepatectomized livers. The diagrams depict the correlation of longitudinal (A) and transverse relaxation (B), diffusion (C) and magnetization transfer (D) with the regenerating liver remnant shown for cPH, ePH, PLF and SHAM.

The increase of T1 relaxation time was significant for animals after ePH compared to cPH (day 1 P < 0.05; day 2 P<0.01; day 3 P<0.001). The ePH group showed significant differences in their relaxation time compared to SHAM operated mice (day 1 - day 3 P<0.001; day 5 P < 0.05), while the T1 relaxation time of the cPH mice does not reach significance compared to SHAM mice **(Fig 4B)**. For T1 relaxation, there was a moderate, but significant negative correlation with the regenerating liver volume for cPH (r = -0,5234; P ≤ 0,0001) and a strong and statistically significant negative correlation for ePH (r = -0,8087, P ≤ 0.0001). In contrast, there is no correlation between T1 relaxation time and the volume of the liver remnant in the case of PLF **(Fig 5A)**.

T2 relaxation time increased rather towards the end of the liver regeneration in all study groups having undergone liver resection **(Fig 4C; Table 1)**. The mice after ePH behaved significantly different as compared to animals after cPH (day 5 P<0.001; day 7 P<0.01) and SHAM (day 3 P<0.01; day 5 P<0.001; day 7 P<0.001), and also the T2 time for cPH was significantly higher than SHAM (day 3 P<0.05; day 5 P<0.05) **(Fig 4C)**. The T2 time of the study groups deviate rather towards the end of the liver regeneration and there was no significant difference for PLF mice allowing differentiation of PLF from ePH and cPH. For T2 relaxation, there was a weak correlation for ePH mice (r = 0,3308, P ≤ 0.05) and no correlation for cPH and PLF mice with the volume of the regenerating liver **(Fig 5B)**.

By comparison to SHAM operated animals, there is a notable decrease of ADC on POD 1 for all study groups having undergone liver resection, most pronounced in the ePH and PLF group **(Fig 4D, Table 2)**. The ADC of the ePH mice recovers again on POD 2, whereas the ADC of the PLF mice continuously diminishes. Comparing the ADC of the ePH group to SHAM and cPH mice, there is a significant difference of ADC (day 1 P<0.05), whereas the cPH group is not significantly different as compared to SHAM. The difference of PLF to SHAM reaches significance (day 3 P<0.01), but there is no significance of PLF to either cPH nor ePH **(Fig 4D)**. Again, there is a weak positive correlation of ADC with the liver volume for cPH (r = 0,3355, P ≤ 0.05), and a moderate positive correlation for ePH (r = 0,4501, P ≤ 0,01) and PLF (r = 0,5911, P ≤ 0,01) **(Fig 5C)**.

Compared to SHAM operated animals, MTR decreases directly after liver resection in all study groups on POD 1. This is especially the case in the two high-risk groups, namely ePH and PLF, which both reach significance (day 1–2 P<0.001; 86% PH day 3 P<0.05) compared to SHAM **(Fig 4E)**. However, albeit their MTR is not significantly different amongst each other, it is statistically significant in comparison to cPH for PLF (day 1 P<0.01; day 2 P<0.001) respectively for ePH (day 1–7 P<0.05). The comparison of MTR for cPH to SHAM does not reach significance **(Fig 4E)**. For MTR, there is a moderate positive correlation for cPH (r = 0,5139, P ≤ 0,001), a strong positive correlation for ePH (r = 0,6915, P ≤ 0.0001) and a very strong positive correlation for PLF with the liver volume (r = 0,8423, P ≤ 0.001) **(Fig 5D)**.

As the measured MR parameters after ePH surgery in isolation do not show a very strong predictive ability for developing PLF, a combinational statistical model using binomial logistic regression has been applied with all MR parameters obtained on day 1 for all animals after ePH, but due to rather small sample size a robust regression model could not be established (P_Omnibus Test_ = 0.292, Nagelkerke R Square = 0.528). Also, noteworthy, all MR parameters retrieved for the baseline measurement prior ePH surgery were statistically assessed and there was clearly no predisposition to develop PLF (P_Omnibus Test_ = 0.560, Nagelkerke R Square = 0.252).

**Fig 6:**
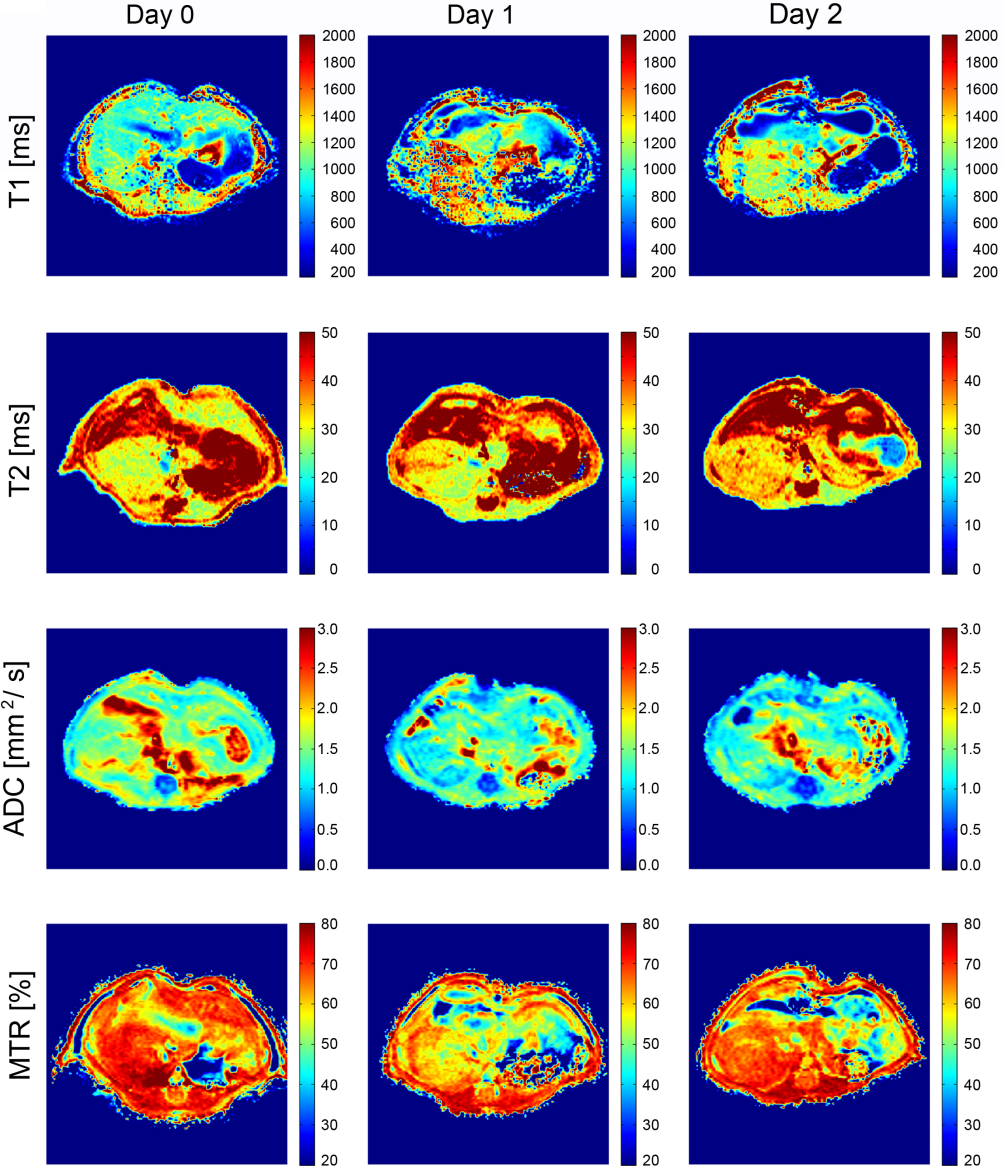
Representative cropped parametric maps of the regenerating liver obtained by voxel-wise fitting of the T1 relaxation time, T2 relaxation time, the parenchymal diffusion represented by the ADC and the magnetization transfer given by MTR for pre-surgical measurement, day 1 and day 2 after conventional partial hepatectomy (cPH). The parametric maps were twofold interpolated.

The alterations of the assessed MR parameters in dependence of the liver volume is further depicted by calculated parametric maps for T1 and T2 relaxation, ADC and MTR for pre-surgical measurement, day 1 and day 2 after liver resection **(Fig 6)**.

## Discussion

Our results show, that multimodal MRI allows for a detailed monitoring of the regenerative process after liver resection. Longitudinal and transverse relaxation MRI as well as DW-MRI and magnetization transfer-MRI are capable of indicating reliably animals suffering from SFSS, but do not permit a robust discrimination of animals with exacerbation towards fatal PLF.

After a considerable amount of liver volume has been removed through surgery, regardless of the exact extent (cPH or ePH), the remaining lobes are required to quickly restore the original liver volume while maintaining their vital function. The majority of liver parenchymal cells are hepatocytes, representing 70% of all liver cells and 80% of the liver weight. Albeit up to 90% of the resting hepatocytes (G0-phase) enter cell proliferation (G1-phase) in almost perfect synchrony immediately after surgery, and in line with our observations, the initially recorded liver remnant volume gain is exclusively attributed to hypertrophy of hepatocytes with an almost twofold increase of cellular volume independent of actual cell division [10]. The replication of cellular content takes place at a later stage (DNA synthesis in mice ~30–40 h post-surgery) (S-phase = DNA synthesis/ G2-phase = protein and organelle synthesis) [11] and the abundance of elevated numbers of binuclear hepatocytes towards the end of the liver regeneration even indicates, that cell division (M-phase = nuclear division (mitosis) with cell division (cytokinesis)) does not directly follow the interphase (i.e. G1 -, S-, G2-phase) [10]. According to a previous study on this mouse model of cPH and ePH, ePH is associated with a delayed progression through the cell cycle, especially with a pronounced procrastination of the M-phase, as compared to cPH [12], which is in line with the representative images depicting immununohistochemistry for the mitotc marker pH3. But also in the H&E stainings, the liver tissue after ePH displayed notable differences compared to cPH, such as a prolonged hypertrophic appearance for ePH accompanied with a seemingly pronounced lipid accumulation of hepatocytes, which may also be reflected by the retrieved MR imaging biomarkers.

The liver volume had increased already after 24 h for both cPH and ePH with the liver remnant volume restored to approximately 50%, respectively 30%.This finding is in keeping with the outlined initial step of liver remnants towards liver regeneration, namely cellular hypertrophy of hepatocytes, being responsible for the instant volume gain after cPH within 24 h after liver resection [10], which apparently also applies to the PLF group due to a volume gain comparable to ePH. Three further investigations on the regenerating mouse liver after resection are in support of these measurements [9], two even applying *in-vivo* MR volumetric assessment [13, 14]. We are unaware of a study using MR volumetry on the liver after ePH in a mouse model, but weight-based data reported by Lehmann et a. [12] suggest a duplication of the liver remnant within 24 h for extended hepatectomy.

These outlined regenerative characteristics on the cellular level are likely to be mirrored by this multimodal MR investigation. Here, T1 and T2 times increase after resection dependent on the extent of the resection with almost complete recovery to baseline towards the end of the regeneration. This finding is in line with previous studies performed on rats also reporting the prolongation of these relaxation times [15–19]. One study conducted on mice assessed T1 relaxation times 5–6 days post PH and does not provide data on liver growth at time of measurement [20]. Comparing studies on rats to our investigation requires caution, nevertheless, two reports using laboratory rats demonstrate a prolongation of T1 times immediately post PH, whereas the T2 time prolongation peaks much later on [17, 19], which is in good agreement with our results. In light of the reported correlation of the elevated parenchymal water content after PH with the increased T1 relaxation times, the observed prolongation of T1 relaxation time appears plausible considering that the tissue water content increases due to the cellular hypertrophy of the hepatocytes. Moreover, the extent of replication initiation of liver cells relies on the extent of the resection [11, 21], thus a higher number of hepatocytes will eventually enter the cell cycle after ePH than compared to cPH, as shown by the expression of cell proliferation markers (Ki67, PCNA) in a study using the same mouse models [12]. Therefore, either the elevated number of hepatocytes undergoing replication or an increased hypertrophy of hepatocytes, or a combination of both, is likely to account for the extended T1 time observed in ePH livers. Potentially due to aborted cell cycle in the case of fatal PLF, the T1 relaxation time deviates on day 2 from mice recovering from SFSS after ePH. This observation might be attributed to the reduced animal number and as a consequence a wider deviation of measured T1 relaxation times would be expected in this cohort. In contrast, on POD 3, the T1 relaxation time increases enormously and surpasses retrieved T1 times of mice after ePH, which eventually survive the surgery. Given the complexity of events during liver regeneration, the delayed prolongation of the T2 time after PH mainly observed in ePH is very difficult to interpret and would be mere conjecture.

The ADC of tissues is affected *in vivo* by cellularity, cell size, cell shape, density and permeability [22] and a recent *in-vitro* investigation indicated, that mainly the cell perimeter length greatly influences the ADC and that especially a raised cell perimeter reduces eventually the retrieved diffusion [23]. On POD 1, the ADC declines considerably in mice after ePH and to a much lesser degree in the animals after cPH and recovers gradually in surviving animals. Again, this finding might be due to the aforementioned hypertrophy of hepatocytes being responsible for a decreased diffusion 24 h post-surgery, which also coincides with the peak of hypertrophy in liver parenchyma post PH [10]. The decline in diffusion is more prominent for liver parenchyma after ePH compared to cPH, and thus potentially attributed to an incomplete progression through the cell cycle. The diffusion is even more restricted in animals developing PLF. In this case, a conceivable explanation might lie within the elevated sinusoidal perfusion causing considerable shear-stress [24] and concomitant congestion of hepatic parenchyma, which inevitably results in vascular and parenchymal damage [25]. An additional factor may be the inadequate venous drainage of the remaining liver parenchyma upon PH, which eventually inflicts hepatic venous congestion [26].

After PH, the MTR decreases in all animals strongly on POD 1 and slowly, but steadily recovers towards the initial MTR prior to surgery. This decrease in MTR is even more pronounced after ePH and in the PLF group than after cPH. Again, these results can be discussed in regard to the hypertrophy and the accompanying unconventional cell division of hepatocytes, as parenchymal cell size increases already after 3h, reaching the maximum 24 h after hepatectomy and resulting in a significantly enlarged cytoplasm without yet having replicated the entire cellular components. Thus, the comparably dilute cytoplasm might provide lesser protein matrix available for magnetization transfer. Moreover, it has long been recognized that liver regeneration upon partial hepatectomy in murine models results in rapid accumulation of intracellular lipids [9, 27–31]. This increased cellular lipid content may additionally be accountable for a further reduction of magnetization transfer. [32]. Thus, either a potentially elevated cellular hypertrophy or a higher number of proliferating, hence swollen hepatocytes, or both, together with a possibly enhanced lipid content might eventually account for the rather pronounced decline in MTR after ePH as compared to cPH. After MTR having eventually reached the minimum, cellular size gradually decreases with progressing mitosis in the liver parenchyma, reducing the amount of hypertrophic cells [10], thus yielding a condensed cytoplasm and allowing again for an increased MTR. There is merely a subtle reduction in MTR after its minimum for PLF, which might be due to a failed progression in the cell cycle.

A sum of these delineated cellular events related to liver regeneration could be causative for the observed MTR changes in the study groups. Another conceivable reason for the decline in MTR might be the inadequate venous drainage of the remaining liver parenchyma upon PH, which eventually inflicts hepatic venous congestion [26]. Still, this would not or not fully explain the relatively distinct time course of MTR alteration in all groups having undergone either cPH or ePH.

Generally, SFSS and PLF are defined as failure of hepatic synthetic and excretory functions such as hyperbilirubinaemia, hypoalbuminaemia, prolonged prothrombin time, elevated serum lactate, and hepatic encephalopathy of different severity [1, 3]. Despite partial hepatectomy being a well-established surgical approach, it still bears the risk of PLF due to insufficient liver volume resulting in SFSS. The prospective evaluation remains difficult [1] and the current lack of an early biomarker for SFSS and PLF precludes an early therapeutic intervention [3, 21, 33].

In this study, only 4 animals in the ePH group developed a fatal PLF as consequence of the inflicted SFSS. Retrospectively, a higher number of ePH mice, resulting potentially in an increased number of fatal PLF would have allowed for a better presentation of MR parameters on this study group. Given the short time granted for animals being assessed by MRI, only a few MR parameters could be elucidated in this investigation. There are more MR-based parameters conceivable, such as arterial spin labelling MRI (ASL-MRI) and MR spectroscopy (MRS), possibly concealing the MR amenable biomarker even being able to predict liver failure and recovery upon SFSS.

Clearly, this multimodal MRI study highlights the capability of *in-vivo* MRI assessments to delineate liver regeneration and to indicate SFSS. The retrieved early biomarkers, predictors for SFSS and potentially for developing PLF, may either represent directly resection related pathologies or regeneration related characteristics. Such non-invasive MRI based predictors might contribute to an improved clinical care for patients with impending SFSS as they may open up new avenues to diagnose SFSS and developing PLF at an early stage eventually providing the prerequisite for an interceptive therapy tailored to patient groups at risk of PLF.

## Ethical approval

This animal study was approved by the local veterinary committee (license no. 131/2011) and was therefore performed in accordance with the applicable international, national and institutional guidelines for the care and use of animals.

## Supporting information

S1 Fig Kaplan-Meier survival curves of mice that underwent MRI examination after 70% cPH, 86% ePH and SHAM. In this study, animals not recovering after ePH constitute a separate group, designated as PLF due to the fatal post-hepatectomy liver failure.
(TIF)

S2 Fig Representative immunohistochemical images depicting hepatocytes positive for phosphorylatedHistone-3 (pH3). The image shows formalin-fixed, paraffin embedded tissue sections of liver parenchyma after cPH on POD 1, 2, 3, 5 and 7 (left panel) and parenchyma after ePH on POD 1, 2, 3, 5 and 7 (right panel).Cells positive for pH3 are indicated by the dark brown stain and cell nuclei are blue. For all images, scale bar is 100 μm.
(TIF)

S3 Fig Representative immunohistochemical images depicting hepatocytes positive for phosphorylatedHistone-3 (pH3). The image shows formalin-fixed, paraffin embedded tissue sections of liver parenchyma after SHAM surgery on POD 1, 2, and 3.Cells positive for pH3 are indicated by the dark brown stain and cell nuclei are blue. For all images, scale bar is 100 μm.
(TIF)

S1 Table Settings of all MRI experiments performed on a 4.7T small animal MRI system (Pharmascan 47/16 US; Bruker BioSpin MRI GmbH, Ettlingen, Germany) with a gradient strength of 375 mT/m and a slew rate of 3375 T/m/s equipped with a linear polarized hydrogen whole-body mouse transmit-receive radiofrequency coil. (PDF)

## Abbreviations

PH: partial hepatectomy
cPH: conventional partial hepatectomy
ePH: extended partial hepatectomy
SFSS: Small-for-Size Syndrome
PLF: post-hepatectomy liver failure
POD: postoperative day
ADC: apparent diffusion coefficient
MT: magnetization transfer
MTR: magnetization-transfer ratio
