# Supplementary figures and images for: Prediction of small for size syndrome after extended hepatectomy: Tissue characterization by relaxometry, diffusion weighted magnetic resonance imaging and magnetization transfer
Source: PLoS One. 2018 Feb 14;13(2):e0192847. doi: 10.1371/journal.pone.0192847 (PMC5812661; doi:10.1371/journal.pone.0192847)

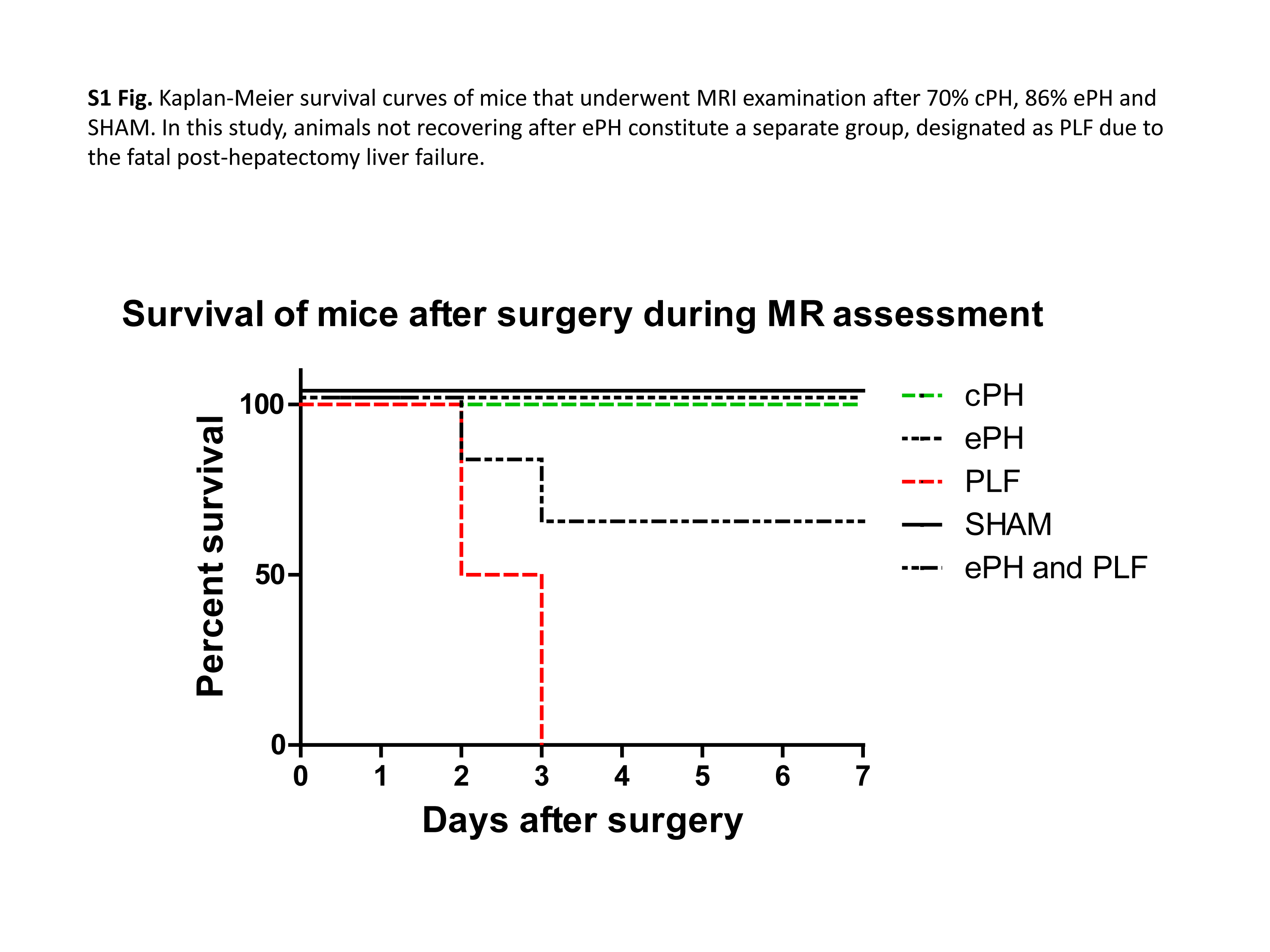

Supplement: S1 Fig — In this study, animals not recovering after ePH constitute a separate group, designated as PLF due to the fatal post-hepatectomy liver failure. (TIF) [file pone.0192847.s001.tif]

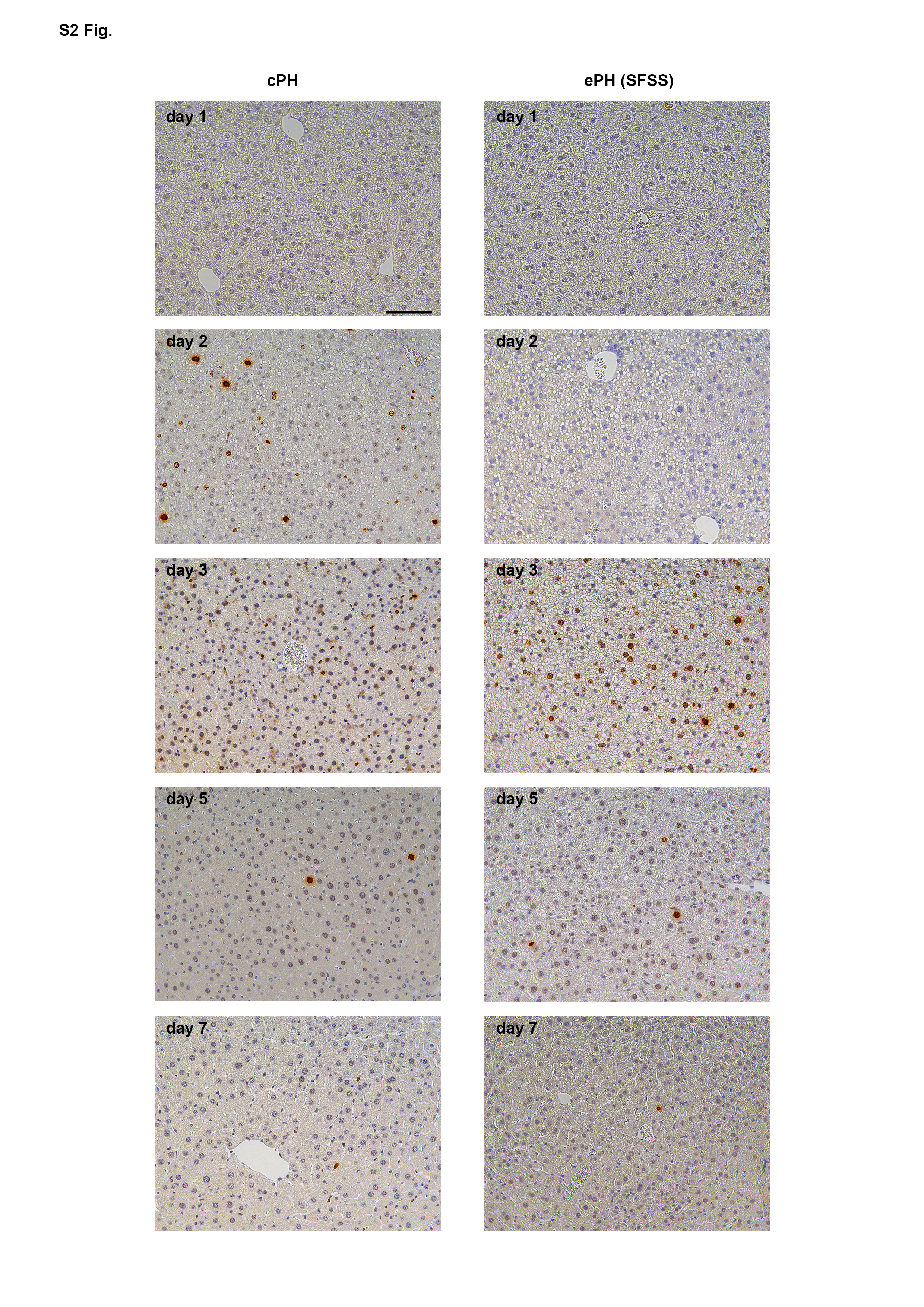

Supplement: S2 Fig — The image shows formalin-fixed, paraffin embedded tissue sections of liver parenchyma after cPH on POD 1, 2, 3, 5 and 7 (left panel) and parenchyma after ePH on POD 1, 2, 3, 5 and 7 (right panel).Cells positive for pH3 are indicated by the dark brown stain and cell nuclei are blue. For all images, scale bar is 100 μm. (TIF) [file pone.0192847.s002.tif]

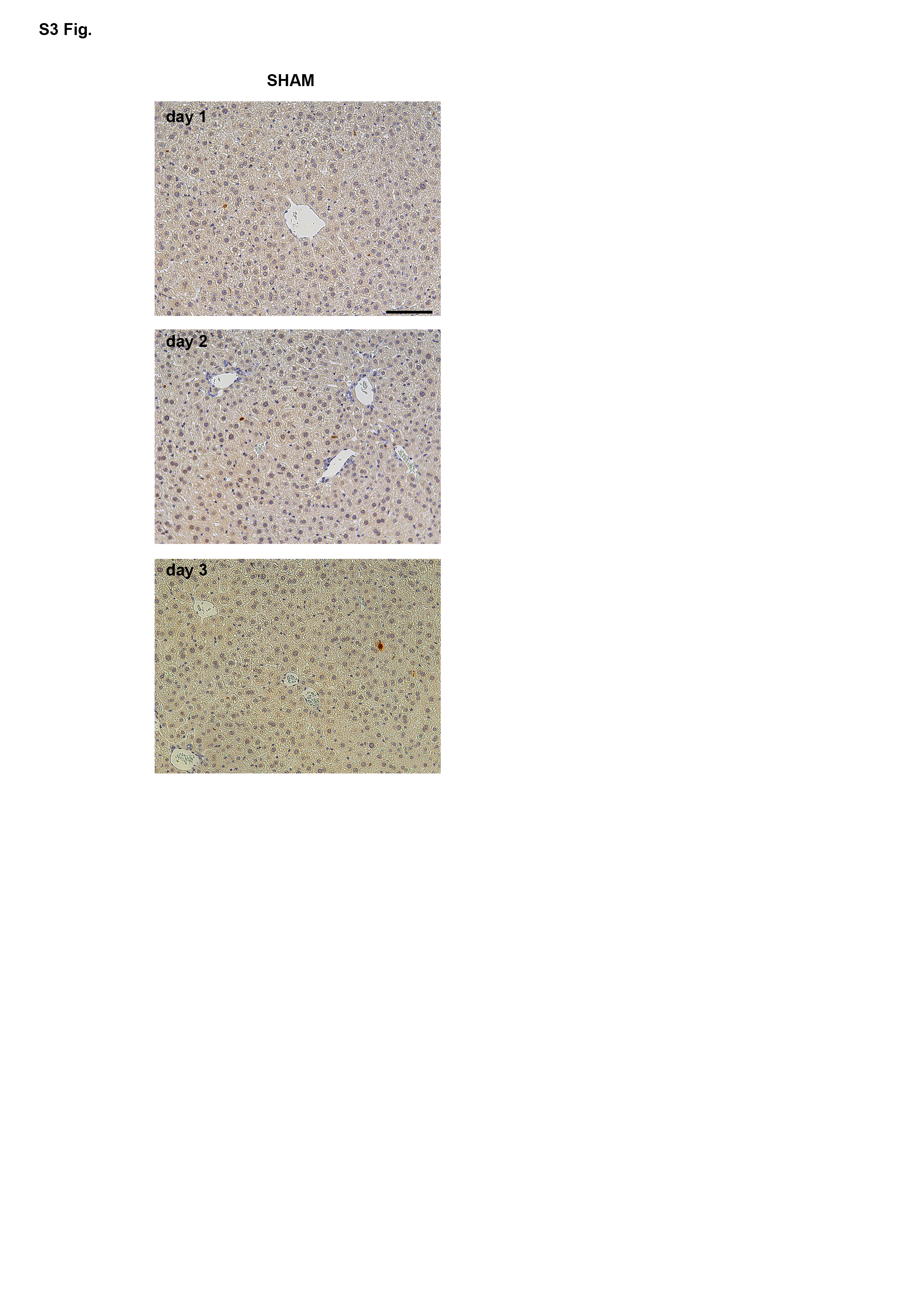

Supplement: S3 Fig — The image shows formalin-fixed, paraffin embedded tissue sections of liver parenchyma after SHAM surgery on POD 1, 2, and 3.Cells positive for pH3 are indicated by the dark brown stain and cell nuclei are blue. For all images, scale bar is 100 μm. (TIF) [file pone.0192847.s003.tif]
