# Supplementary material for: Prediction of small for size syndrome after extended hepatectomy: Tissue characterization by relaxometry, diffusion weighted magnetic resonance imaging and magnetization transfer
Source: PLoS One. 2018 Feb 14;13(2):e0192847. doi: 10.1371/journal.pone.0192847 (PMC5812661; doi:10.1371/journal.pone.0192847)
Supplement: S1 Table — (PDF) [file pone.0192847.s004.pdf]

S1 Table. Settings of all MRI experiments performed on a 4.7T small animal MRI system (Pharmascan 47/16 US; Bruker BioSpin MRI GmbH, Ettlingen, Germany) with a gradient strength of 375 mT/m and a slew rate of 3375 T/m/s equipped with a linear polarized hydrogen whole-body mouse transmit-receive radiofrequency coil.

MR parameter	3D-FLASH (Volumetry)	2D-trueFISP	RARE (RAREVTR)	EPI (DTI-EPI)	3D-FLASH (MTR)
TE	2.6 ms	2.25 ms	11, 33, 55, 77, 99 ms	30 ms	2.65 ms
TR	15.0 ms	4.5 ms	118, 258, 400, 800, 1500, 3000, 4000 ms	3000 ms	18.4 ms
AVG	4	4	2	8	8
FA	20°	60°	180°	12°	12°
FoV	30 x 30 mm	30 x 30 mm	30 x 30 mm	30 x 30 mm	30 x 30 mm
Slice thickness; Number of slices	22.5 mm; 1 slice	1.5 mm; 1 slice	1.5 mm; 10 slices	1.5 mm; 10 slices	15 mm; 1 slice
Matrix	256 x 256 x 96	128 x 128	128 x 128	128 x 128	128 x 128 x 10
Voxel	0.117 x 0.117 x 0.234 mm	0.234 x 0.234 x 1.5 mm	0.234 x 0.234 x 1.5 mm	0.234 x 0.234 x 1.5 mm	0.234 x 0.234 x 1.5 mm
BW	100 000 Hz	81522 Hz	480769 Hz	250 000 Hz	100 000 Hz
MagTrans	n/a	n/a	n/a	n/a	on/ off
Trigger Module	off	on	on	on	off
Fat suppression	off	off	off	on	off
Remarks	n/a	n/a	RARE factor = 2	1 diffusion direction, 2 b-values (b =0; b =800 s/mm^2)	MT-prepulse off-resonance 1500 Hz; FA = 1500; pulse length = 10.96 ms; BW = 250Hz, interpulse delay 0.01 ms
